# MicroRNA-100 is a potential molecular marker of non-small cell lung cancer and functions as a tumor suppressor by targeting polo-like kinase 1

**DOI:** 10.1186/1471-2407-12-519

**Published:** 2012-11-14

**Authors:** Jing Liu, Kai-Hua Lu, Zhi-Li Liu, Ming Sun, Wei De, Zhao-Xia Wang

**Affiliations:** 1 Department of Oncology, The Second Affiliated Hospital of Nanjing Medical University, 121 Jiangjiayuan Road, Nanjing, Jiangsu, 210011, Peoples Republic of China; 2 Immunology and Reproductive Biology Lab of Medical School and State Key Laboratory of Pharmaceutical Biotechnology, Nanjing University, Nanjing, Jiangsu, 210093, Peoples Republic of China; 3 Department of Oncology, The First Affiliated Hospital of Nanjing Medical University, Nanjing, Jiangsu, 210029, Peoples Republic of China; 4 Department of Biochemistry and Molecular Biology, Nanjing Medical University, Nanjing, Jiangsu, 210029, Peoples Republic of China

## Abstract

**Background:**

Polo-like kinase 1 (PLK1) is highly expressed in many human cancers and regulates critical steps in mitotic progression. Previously, we have reported that PLK1 was overexpressed in non-small cell lung cancer (NSCLC), but the underlying molecular mechanisms are not well understood. By using microRNA (miR) target prediction algorithms, we identified miR-100 that might potentially bind the 3’-untranslated region of PLK1 transcripts. The purpose of this study was to investigate the roles of miR-100 and its association with PLK1 in NSCLC development.

**Methods:**

Taqman real-time quantitative RT-PCR assay was performed to detect miR-100 expression 10 NSCLC tissues and corresponding nontumor tissues. Additionally, the expression of miR-100 in 110 NSCLC tissues and its correlation with clinicopathological factors or prognosis of patients was analyzed. Finally, the effects of miR-100 expression on growth, apoptosis and cell cycle of NSCLC cells by posttranscriptionally regulating PLK1 expression were determined.

**Results:**

MiR-100 was significantly downregulated in NSCLC tissues, and low miR-100 expression was found to be closely correlated with higher clinical stage, advanced tumor classification and lymph node metastasis of patients. The overall survival of NSCLC patients with low miR-100 was significantly lower than that of those patients with high miR-100, and univariate and multivariate analyses indicated that low miR-100 expression might be a poor prognostic factor. Also, miR-100 mimics could lead to growth inhibition, G_2_/M cell cycle arrest and apoptosis enhancement in NSCLC cells. Meanwhile, miR-100 mimics could significantly inhibit PLK1 mRNA and protein expression and reduce the luciferase activity of a PLK1 3’ untranslated region-based reporter construct in A549 cells. Furthermore, small interfering RNA (siRNA)-mediated PLK1 downregulation could mimic the effects of miR-100 mimics while PLK1 overexpression could partially rescue the phenotypical changes of NSCLC cells induced by miR-100 mimics.

**Conclusions:**

Our findings indicate that low miR-100 may be a poor prognostic factor for NSCLC patients and functions as a tumor suppressor by posttranscriptionally regulating PLK1 expression.

## Background

Lung cancer is the leading cause of cancer-related deaths around the world, among both men and women, with an incidence of over 200000 new cases per year and a very high mortality rate [1]. Approximately 85% of all lung cancer cases are categorized as non-small cell lung cancer (NSCLC). Despite much progress in early detection and treatment, the 5-year survival rate for NSCLC patients at later stages is only 5-20% [2]. Thus, a better understanding of the molecular mechanisms underlying NSCLC progression and development will be helpful for improvement of current therapeutics and the identification of novel targets.

PLK1 belongs to a family of conserved serine/threonine kinases that are involved in cell-cycle progression and various mitotic stages [3]. The overexpression of PLK1 has been reported to play critical roles in malignant transformation and tumor development [4,5]. It has been found that PLK1 is overexpressed in a variety of human tumours and has prognostic potential in cancer, indicating its involvement in carcinogenesis and its potential as a therapeutic target [6]. Although Wolf and his colleagues found that PLK mRNA expression provided a new independent prognostic indicator for patients with NSCLC [7], the clinical significance of PLK1 protein in NSCLC was unclear. In our previous study, we have shown that high PLK1 protein expression was significantly correlated with higher clinical stage, advanced tumor classification and lymph node metastasis of NSCLC patients and might be a poor prognostic molecular marker [8]. Meanwhile, we also found that RNA interference-mediated PLK1 downregulation could inhibit in vitro and in vivo proliferation, induce cell arrest of G_2_/M phase, increase apoptosis and enhance chemo-or radiosensitivity of NSCLC cells. In addition, Spänkuch-Schmitt B’ et al. reported that downregulation of human polo-like kinase activity by antisense oligonucleotides induced growth inhibition in cancer cells including NSCLC cell line (A549) [9]. This research group also found that PLK1 function appeared to be essential for centrosome-mediated microtubule events and, consequently, for spindle assembly and siRNAs targeted against human PLK1 might be valuable tools as antiproliferative agents against a broad spectrum of neoplastic cells including NSCLC cell line (A549) [10]. Raab and his colleagues found that the primary cells’proliferation, spindle assembly and apoptosis exhibited only a low dependency on Plk1 in contrast to the addiction of many cancer cell lines to the non-oncogene Plk1 [11]. Also, Liu and colleagues showed that normal cells but not cancer cells could survive severe Plk1 depletion [12]. These data further support suggestions that Plk1 might be a feasible cancer therapy target. However, the molecular mechanisms of PLK1 upregulation in NSCLC are still unclear. MicroRNAs are a class of single-stranded RNA molecules of 21–23 base pair in length and regulate target genes expression through specific base-pairing interactions between miRNA and untranslated regions of targeted mRNAs [13]. miRNAs can bind to the 3’-untranslated regions (UTRs) of target mRNAs, which leads to mRNA degradation or repression of mRNA translation. It has been reported that approximately > 30% of protein-coding genes can be directly modulated by miRNAs [14]. Other groups have shown that underexpressed miR-100 leads to Plk1 overexpression, which in turn contributes to nasopharyngeal cancer progression [15]. It was also reported that miR-100 could affect the growth of epithelial ovarian cancer cells by post-transcriptionally regulating polo-like kinase 1 expression [16]. However, the status of miR-100 expression in NSCLC is unclear, and whether miR-100 plays a critical role in NSCLC development by posttranscriptionally regulating PLK1 expression needs to be further elucidated.

In the present study, we set out to detect the expression of miR-100 in NSCLC tissues and analyze its correlation with clinicopathological factors or prognosis of NSCLC patients, and post-transcriptional regulatory relation between miR-100 and PLK1 in NSCLC cells, which will provide one day a potential molecular therapeutic target for human NSCLCs.

## Methods

### Sample population

A total of 112 primary NSCLCs were collected from the Department of Cardiothoracic Surgery, Nanjing Medical University between 2003 and 2006. All patients gave written informed consent. The study was approved by the Ethic Committee of Second Affiliated Hospital, Nanjing Medical University and it was performed in compliance with the Helsinki Declaration. All patients did not receive chemotherapy or radiotherapy prior to surgery. Patient characteristics are shown in Additional file 1 Table S1. Disease histology was determined in accordance to the criteria of the World Health Organization. Pathologic staging was performed in accordance to the current International Union Against Cancer tumor-lymph node-metastasis classification. 10 randomly selected NSCLC tumors and their matched histologically normal lung parenchyma adjacent to the tumors (within 1 cm of the discrete tumor margin) were immediately frozen in liquid nitrogen and stored at −70°C until use. All tissue samples were snap-frozen in liquid nitrogen, which were transferred to 500 ml TRIzol solution (Invitrogen, CA, USA) immediately after harvesting in order to avoid mRNA degradation.

### Cell line and cell culture conditions

NSCLC cell line (A549) was cultured in Dulbecco’s modified Eagle’s medium (Invitrogen, Carlsbad, CA) supplemented with 10% fetal bovine serum, 100 U/ml penicillin and 100 μg/ml streptomycin. Cell cultures were incubated in a humidified atmosphere of 5% CO_2_ at 37°C.

### Construction of plasmid vectors

Previously, the pcDNA/PLK1 vector with the PLK1 coding region was successfully constructed and conserved by our lab. To construct a luciferase reporter vector, the PLK1 3’-UTR fragment containing putative binding sites for miR-100 was amplified by PCR using the following primers: sense 5’-*CCATACTGGTTGGCTCCCGCGG*-3’ and reverse 5’-*ATGTGCATAAAGCCAAGGAAAGG*-3’, and inserted into downstream of the luciferase gene in the pLuc luciferase vector (Ambion, USA) and named PLK1 3’-UTR-wild. Site-directed mutagenesis of the miR-100 target-site in the PLK1-3’-UTR was performed using the Quick-change mutagenesis kit (Stratagene, Heidelberg, Germany) and named PLK1 3’-UTR-mut according to the manufacturer’s instructions.

### Transfection of miR-100 mimics or inhibitor

MiR-100 mimics (or anti-miR-100) and their negative control oligonucleotides (miR-NC or anti-miR-100) were obtained from Ambion Inc (Austin, TX, USA). siRNA/PLK1 (5’-*AAGGGCGGCUUUGCCAAGUGC*-3’) and its negative control oligonucleotide (siRNA/NC: 5’-*AAUUUGGCCGGGCCGUGCG*-3’) were purchased from Santa Cruze Inc (CA, USA). The transfection were performed using Lipofectamine™ 2000 (Invitrogen, USA) according to the instructions provided by the manufacturer. The transected cells were resuspended and cultured in regular culture medium for 48-72 h before analysis.

### TaqMan RT-PCR for miRNA quantification

Total RNA was isolated from the cell lines with Trizol™ (Invitrogen, USA), reverse transcribed using Taqman™ microRNA reverse transcription kit and subjected to real-time PCR using TaqMan™ MicroRNA Assay kit (Applied Biosystems, USA) according to the manufacturer’s instructions. Reactions were performed using Stratagene Mx3000 instrument in triplicate. MiRNA expression was normalized to U6.

### Semi-quantitative RT-PCR assay

Total RNA isolated from cells or tissues using an RNeasy Mini Kit (Qiagen, USA) was reverse-transcribed with random hexamers and a Transcriptor First Strand cDNA Synthesis Kit (TaKaRa, Dalian, China). The GAPDH primers were as follows: sense: 5’-*CACCATCTTCCAGG-AGCGAG*-3’, reverse: 5’-*TCACGCCACAGTTTCCCGGA*-3’ (372 bp). Equal cDNA amounts from each sample were amplified using the following primers to detect PLK1 expression: sense, 5’-*TTCGTGTTCGTGGTGTTGGA*-3’; reverse, 5’-*CTCGTCATTAAGCAGCTCGT*-3’ (563 bp). Thermal cycles were: 1 cycle of 94°C for 3 min; 30 cycles of 94°C for 40s, 56°C for 40s, 68°C for 90s; followed by 72°C for 10 min. RT-PCR products were electrophoresed in a 1.5% agarose gel with ethidium bromide staining.

### Western blot assay

Proteins were isolated and separated by 7.5% SDS–PAGE and electrotransferred to polyvinylidene fluoride membranes. Residual binding sites on the membrane were blocked in 5% skim milk for 1 h at room temperature. The blots were incubated with primary antibodies against human PLK1 (Santa Cruz, CA, USA) at 1:500 overnight at 4°C and then with anti-rabbit IgG (horseradish peroxidase-conjugated secondary antibody) for 1 h at room temperature. After washing, the membranes were developed with an ECL plus Western blotting detection system (Amersham).

### 3-(4,5-dimethylthazol-2-yl)-2,5-diphenyltetrazolium bromide (MTT) assay

The transfected cells (A549) were seeded into 96-well culture plates. After overnight incubation, cells treated with various concentrations of chemotherapeutic drugs. Following incubation for 24 h, cell growth was measured following addition of 0.5 mg/ml 3-(4,5-dimethyl-thia-zol-2-yl)-2,5-diphenyltetrazoliumbromide (MTT, Sigma) solution. About 4 h later, the medium was replaced with 100 μL dimethylsulfoxide (DMSO, Sigma) and vortexed for 10 min. Absorbance (A) was then recorded at 490 nm using a microplate reader (Bio-Rad, USA).

### Hoechst staining assay

Cells were cultured in 6 well plates to confluence and Hoechst 33342 (Sigma, USA) was then added. Nuclear morphology changes were detected by fluorescence microscopy using a filter for Hoechst 33342 (365 nm). The percentages of Hoechst-positive nuclei per optical field (≥ 50 fields) were counted.

### Flow cytometric analysis of cell cycle

Cells were harvested at 70% confluence and fixed in 70% ethanol at −20°C. After a PBS wash, cells were treated with RNase A at 37°C for 30 min. After centrifugation, cells were resuspended in propidium iodide (PI) (50 μg/ml) for 30 min at room temperature. DNA content was then evaluated using a FACScan flow cytometer (Becton Dickinson, Mountain View, CA).

### Luciferase assay

The constructs were sequenced and named pLuc-PLK1-wt or pLuc-PLK1-mut. For reporter assays, A549 cells were cultured in 24-well plates and each transfected with 100 ng of pLuc-PLK1-wt or pLuc-PLK1-mut and 50 nM of miR-100 mimics or anti-miR-100 using Lipofectamine 2000 (Invitrogen, USA). Forty eight hours after transfection, cells were harvested and assayed with Dual-Luciferase Reporter Assay kit (Promega, USA) according to the manufacturer’s instructions.

### Statistical analysis

Data are presented as mean ± SD. For comparison of means between two groups, a two-tailed *t*-test was used, and for comparison of means among three groups, one-way ANOVA was used The Spearman correlation test was used for analyses of primary tumors. Survival probabilities were determined using Kaplan-Meier analysis and the significance of difference was analyzed by a log-rank test. Significance was accepted at *P* < 0.05.

## Results

### MiR-100 was significantly downregulated in NSCLC tissues compared with corresponding nontumor tissues

**Figure 1:**
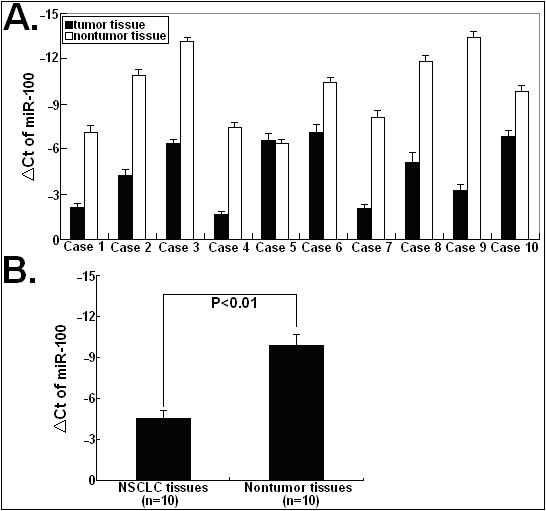
**Taqman real-time quantitative analysis of miR-100 expression in tissue samples.** (**A**) Determining the ΔCt values of miR-100 in 10 cases of NSCLC tissues and corresponding nontumor tissues. (**B**) Comparison of mean ΔCt of miR-100 between 10 cases of NSCLC tissues (−4.527 ± 1.04) and corresponding nontumor tissues (−9.878 ± 1.33; *P* < 0.01). The mean and standard deviation of expression levels relative to U6 expression levels are shown and are normalized to the expression in the normal tissue of each matched pair. All experiments were performed at least in triplicate. Corresponding *P* values analyzed by *t*-tests are indicated.

Real-time quantitative RT-PCR assay was performed to detect the expression of miR-100 in 10 NSCLC tissues and corresponding nontumor tissues. As shown in Figure 1A, the relative level of miR-100 expression was significantly lower in NSCLC tissues than in corresponding nontumor tissues. The mean miR-100 expression level (△Ct) was −4.527 ± 1.04 for NSCLC tissues (n = 10) and −9.878 ± 1.33 for corresponding nontumor tissues (n = 10); this difference was statistically significant with a *P*-value of <0.01 (Figure 1B). Thus, it was concluded that downregulation of miR-100 might play important roles in lung tumorigenesis.

### Association of miR-100 expression with clinicopathological features of NSCLC patients

Real-time quantitative RT-PCR assay was performed to detect the expression of miR-100 in 110 NSCLC tissues, and the association of miR-100 expression with clinicopathological features of NSCLC patients was performed (Additional file 1 Table S1). The mean miR-100 expression level (△Ct) was −6.218 ± 1.53 for NSCLC tissue (n = 110). Patients with miR-100 expression (△Ct < −6.218) were considered as the low expression group (n = 64), while patients with miR-100 expression (△Ct ≥ −6.218) were considered as the high expression group (n = 46). By statistical analyses, we showed that low miR-100 expression was closely correlated with higher clinical stage, advanced tumor classification and lymph node metastasis (*P* = 0.005, 0.013 and 0.001, respectively). However, the expression of miR-100 was not correlated with other factors of patients including sex, age, smoking, histological type (*P* = 0.488, 0.583, 0.359 and 0.871, respectively). These data showed that low miR-100 expression might play important roles in NSCLC development.

### Association of miR-100 with prognosis of NSCLC patients

**Figure 2:**
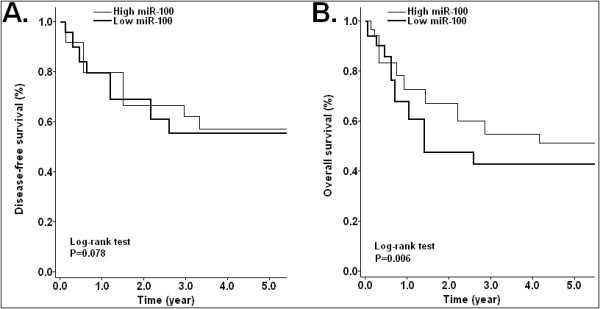
**Kaplan-Meier survival curves of NSCLC patients.** Kaplan-Meier survival curves for NSCLC patients based on the median level of fold change. The P-value was calculated using the log-rank test between patients with high- and low-fold changes. Disease-free or overall survival of patients with high vs. low miR-100 expression levels are shown. (**A**) The 5-year disease-free survival rate showed no difference between NSCLC patients with high miR-100 and those with low miR-100 (*P* = 0.078). (**B**) The 5-year overall survival rate of NSCLC patients with high miR-100 was significantly higher than that of those patients with low miR-100 (*P* = 0.006). *P* < 0.05 indicates a significant difference between groups. Corresponding *P* values analyzed by log-rank tests are indicated.

The association of miR-100 expression with prognosis of NSCLC patients was also investigated by Kaplan-Meier analysis and log-rank test. As shown in Figure 2A, there was no significant difference in 5-year disease-free survival (DFS) between patients with low miR-100 expression and those with high miR-100 expression (*P* = 0.078). However, the 5-year overall survival (OS) of patients with high miR-100 expression was significantly higher than that of those with low miR-100 expression (*P* = 0.006; Figure 2B). Univariate analysis showed that clinical stage, lymph node metastasis and low miR-100 expression were significantly correlated with poor over survival of NSCLC patients (*P* = 0.003, 0.016 and 0.022, respectively; Table 1). Multivariate analysis using the Cox proportional hazard model indicated that the status of lymph node metastasis and the level of miR-100 expression were independent prognostic factors for NSCLC patients (*P* = 0.036 and 0.008, respectively; Table 1). Thus, miR-100 expression could affect the prognosis of NSCLC patients, and low miR-100 expression might be a poor prognostic factor.

**Table 1:** Univariate and multivariate analysis of prognostic factors by Cox regression analysis

**Clinicopathological features**	**Univariate analysis**		**Multivariate analysis**	
	**RR (95% CI)**	***P*-value**	**RR (95% CI)**	***P*-value**
Male (Male / female)	2.27 (0.87-3.11)	0.108	1.99 (0.67-2.63)	0.112
Age (year) (>60 / ≤60)	1.42 (0.92-1.79)	0.321	2.56 (0.89-3.12)	0.304
Smoking (Smoker / Nonsmoker)	1.65 (0.58-1.88)	0.084	2.13 (0.75-2.70)	0.156
Histological type (SCC / AD)	2.08 (0.39-2.75)	0.202	0.87 (0.46-1.68)	0.286
Clinical stage (III / I + II)	2.76 (1.23-3.82)	0.003	3.13 (0.95-4.03)	0.067
Tumor classification (T_3+4_ / T_1+2_)	0.98 (0.44-1.55)	0.215	1.53 (0.74-1.88)	0.082
Lymph node metastasis (N_1+2_ / N_0_)	3.12 (2.03-4.12)	0.016	1.65 (1.12-3.58)	0.036
miR-100 expression (Low / High)	1.74 (1.04-2.36)	0.022	2.44 (1.89-2.95)	0.008

RR: relative ratio; 95% CI: 95% confidence interval.

SCC: squamous cell carcinoma; AD: adenocarcinoma.

### Effects of miR-100 expression on growth, apoptosis and cell cycle of NSCLC cells

**Figure 3:**
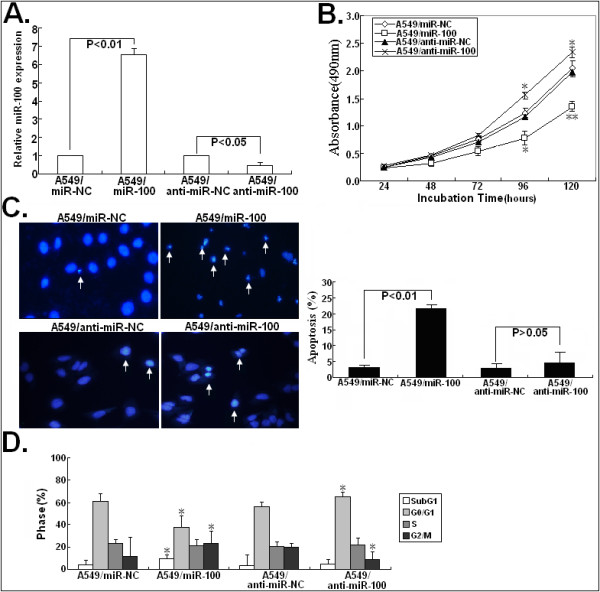
**Effects of miR-100 mimics or inhibitor on growth, apoptosis and cell cycle of NSCLC cells.** (**A**) 48h after A549 cells were transfected with miR-100 mimics (or miR-NC mimics) or anti-miR-100 (or anti-miR-NC), Taqman real-time quantitative RT-PCR analysis of miR-100 expression. (**B**) MTT analysis of growth in A549/miR-100 (or A549/miR-NC) or A549/anti-miR-100 (or A549/anti-miR-NC) cells. (**C**) Hoechst staining analysis of apoptosis in A549/miR-100 (or A549/miR-NC) or A549/anti-miR-100 (or A549/anti-miR-NC) cells. Fragmentation of the nucleus into oligonucleosomes and chromatin condensation were examined by fluorescence microscopy. The percentage of Hoechst-positive nuclei per optical field (at least 50 fields) was counted. (**D**) Flow cytometric analysis of cell cycle distribution in A549/miR-100 (or A549/miR-NC) or A549/anti-miR-100 (or A549/anti-miR-NC) cells. * and ** indicate *P* < 0.05 and *P* < 0.01, respectively. Each experiment was performed at least in triplicate. Corresponding *P* values analyzed by *t*-tests are indicated.

Next, the effects of miR-100 expression on malignant phenotypes of NSCLC cells were investigated. 48 h after transfection with miR-100/mimics (or miR-NC/mimics) or anti-miR-100 (or anti-miR-NC), Real-time quantitative RT-PCR assay was performed to detect the expression of miR-100 in A549 cells. As shown in Figure 3A, the level of miR-100 expression in A549/miR-100 cells was significantly increased by approximately 586.7% compared with A549/miR-NC cells (*P* < 0.01). Compared with that in A549/anti-miR-100 cells, the level of miR-100 expression in A549/anti-miR-100 cells was also significantly decreased by approximately 54.3% (*P* < 0.05). The results of MTT assay showed that miR-100 mimics could markedly inhibit the growth of A549 cells while miR-100 inhibitors could slightly promote the growth of A549 cells (Figure 3B). Then, we analyzed the effect of miR-100 expression on apoptosis of NSCLC cells (Figure 3C). The results of Hoechst staining assay showed that the apoptotic rate of A549/miR-100 cells was significantly increased by 18.3 ± 1.4% compared with A549/miR-NC cells (*P* < 0.01). However, the apoptotic rate of A549/anti-miR-100 cells showed no significance changes compared with that of A549/anti-miR-NC cells (*P* > 0.05). Next, the effect of miR-100 expression on cell cycle of A549 cells was also determined by flow cytometry (Figure 3D). Compared with A549/miR-NC cells, A549/miR-100 cells showed the increased percentage of apoptotic cells (SubG_1_) and G_2_/M stage cells and the decreased percentage of G_0_/G_1_ stage cells (*P* < 0.05). However, the percentage of S stage cells showed no difference between those two transfected A549 cells. Compared with A549/anti-miR-NC cells, A549/anti-miR-100 cells showed the decreased percentage of G_2_/M stage cells and the increased percentage of G_0_/G_1_ stage cells (*P* < 0.05). Likewise, the percentage of S stage cells showed no difference between A549/anti-miR-NC and A549/anti-miR-100 cells. From these data, it was concluded that miR-100 could inhibit growth of NSCLC cells by modulating apoptosis and cell-cycle distribution.

### PLK1 is a functional target of miR-100 in NSCLC

**Figure 4:**
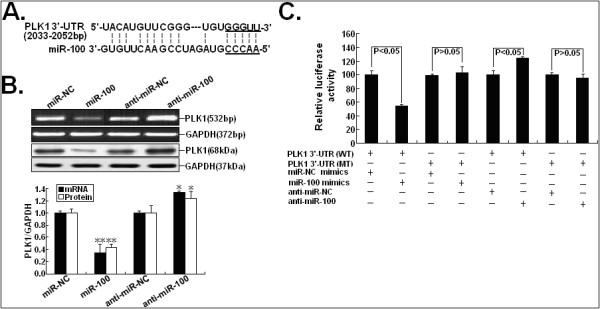
**PLK1 is a direct target of miR-100.** (**A**) Alignment between the predicted miR-100 target sites and miR-100 is shown. (**B**) RT-PCR and Western Blot assays were performed to detect the expression of PLK1 mRNA and protein expression in A549 cells transfected with miR-100 mimics (miR-NC mimics) or anti-miR-100 (anti-miR-NC). (**C**) A549 cells were co-transfected with miR-100 mimics or anti-miR-100 and pLUC vector with PLK1 3’-UTR-wild or mut. After 24 hours, the luciferase activity was measured. Values are presented as relative luciferase activity after normalization to Renilla luciferase activity. * and ** indicate *P* < 0.05 and *P* < 0.01, respectively. The data are expressed as the mean value ± SEM of the results obtained from three independent experiments. Corresponding *P* values analyzed by *t*-tests are indicated.

It has been reported that miRNAs can function post-transcriptionally by reducing protein yield from specific target mRNAs. To identify miR-100 targets, we performed in-silico screening using TargetScan with a recently described strategy [17]. We found that, among the predicted miR-100 target genes, the 3’-UTR of PLK1 gene contained binding sites for miR-100 with reasonable scores. In other cancers, PLK1 has been reported to be a functional target of miR-100. Sequence analyses revealed that the 3’-UTR of PLK1 mRNA contains a putative site partially complementary to miR-100 (Figure 4A). To experimentally validate whether PLK1 is a possible target of miR-100 in NSCLC, we detected the expression of PLK1 mRNA and protein in miR-100 mimics or anti-miR-100-transfected A549 cells. RT-PCR and Western Blot assays showed that the expression levels of PLK1 both mRNA and protein were significantly downregulated by miR-100 mimics while miR-100 inhibitors could increase the expression levels of PLK1 mRNA and protein in A549 cells (Figure 4B). To further determine whether PLK1 was a bona fide target of miR-100-mediated gene overexpression, the entire 3’-UTR of PLK1 mRNA and the miR-100 binding region within 3’-UTR of PLK1 mRNA were cloned into a luciferase reporter. As shown in Figure 4C, upregulation of miR-100 could result in a significant decrease in luciferase activity when the reporter contained a wild-type sequence (WT), but not when it contained a mutant sequence (MT) within the miR-100 binding site (five nucleotides within the complementary seed sequence). Meanwhile, downregulation of miR-100 could lead to a significant increase in luciferase activity the reporter contained a wild-type sequence (WT), but not when it contained a mutant sequence (MT) within the miR-100 binding site. Taken together, these data indicate that miR-100 directly targets PLK1 in NSCLC cells.

### Effects of siRNA-mediated PLK1 inhibition on malignant phenotypes of NSCLC cells

**Figure 5:**
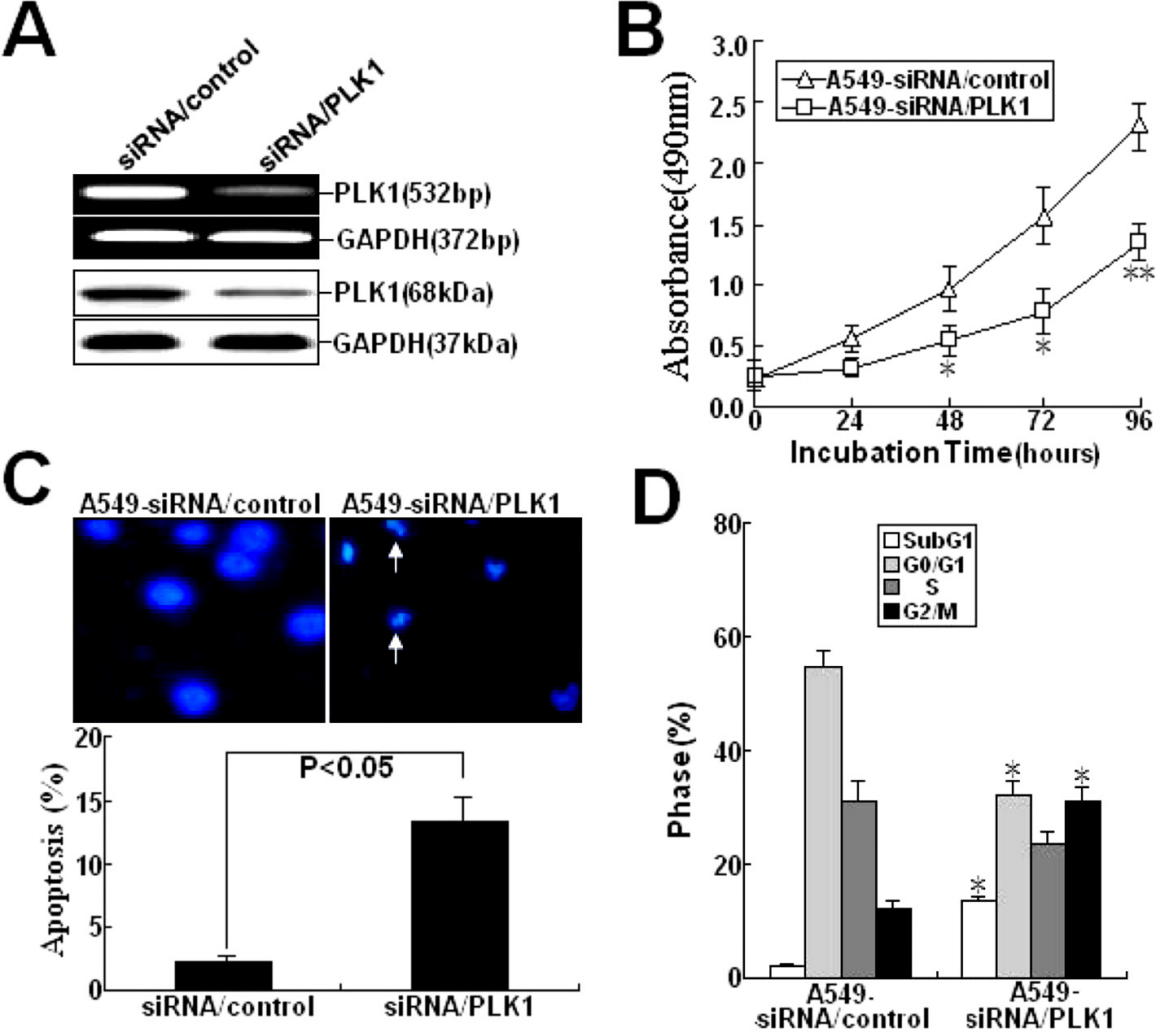
**Effects of siRNA targeting PLK1 on growth, apoptosis and cell cycle of NSCLC cells.** (**A**) RT-PCR and Western Blot analysis of PLK1 mRNA and protein expression in A549-siRNA/PLK1 or A549-siRNA/control cells. (**B**) MTT analysis of growth in A549 cells at different time points (0, 24, 48, 72 or 96h) after transfection with siRNA/PLK1 or siRNA/control. (**C**) Hoechst staining analysis of apoptosis in A549/miR-100 or A549/miR-NC cells. The percentage of Hoechst-positive nuclei per optical field (at least 50 fields) was counted. (**D**) Flow cytometric analysis of cell cycle distribution in A549-siRNA/PLK1 or A549-siRNA/control cells. * and ** indicate *P* < 0.05 and *P* < 0.01, respectively. Each experiment was performed at least in triplicate. Corresponding *P* values analyzed by *t*-tests are indicated.

\Next, siRNA targeting PLK1 was used to downregulate PLK1 expression and analyze its effects on phenotypes of NSCLC cells including growth, apoptosis and cell cycle. As shown in Figure 5A, the expression levels of PLK1 mRNA and protein were significantly downregulated in A549-siRNA/PLK1 cells compared with A549-siRNA/control cells. MTT assay indicated that the growth rate of A549-siRNA/PLK1 cells was significantly lower than that of A549-siRNA/control cells (Figure 5B). Hoechst staining assay showed that the apoptotic rate of A549-siRNA/PLK1 cells was significantly increased by approximately 12.3% compared with that of A549-siRNA/control cells (Figure 5C). Finally, flow cytormetric analysis of cell cycle showed that A549-siRNA/PLK1 cells showed the increased percentage of apoptotic cells and G_2_/M stage cells and the decreased percentage of G_0_/G_1_ stage cells compared with A549-siRNA/control cells (Figure 5D). Therefore, siRNA-mediated downregulation of PLK1 could mimic the effects of increased miR-100 in NSCLC cells.

### Overexpression of PLK1 could rescue the effects of ectopic miR-100 expression in NSCLC cells

**Figure 6:**
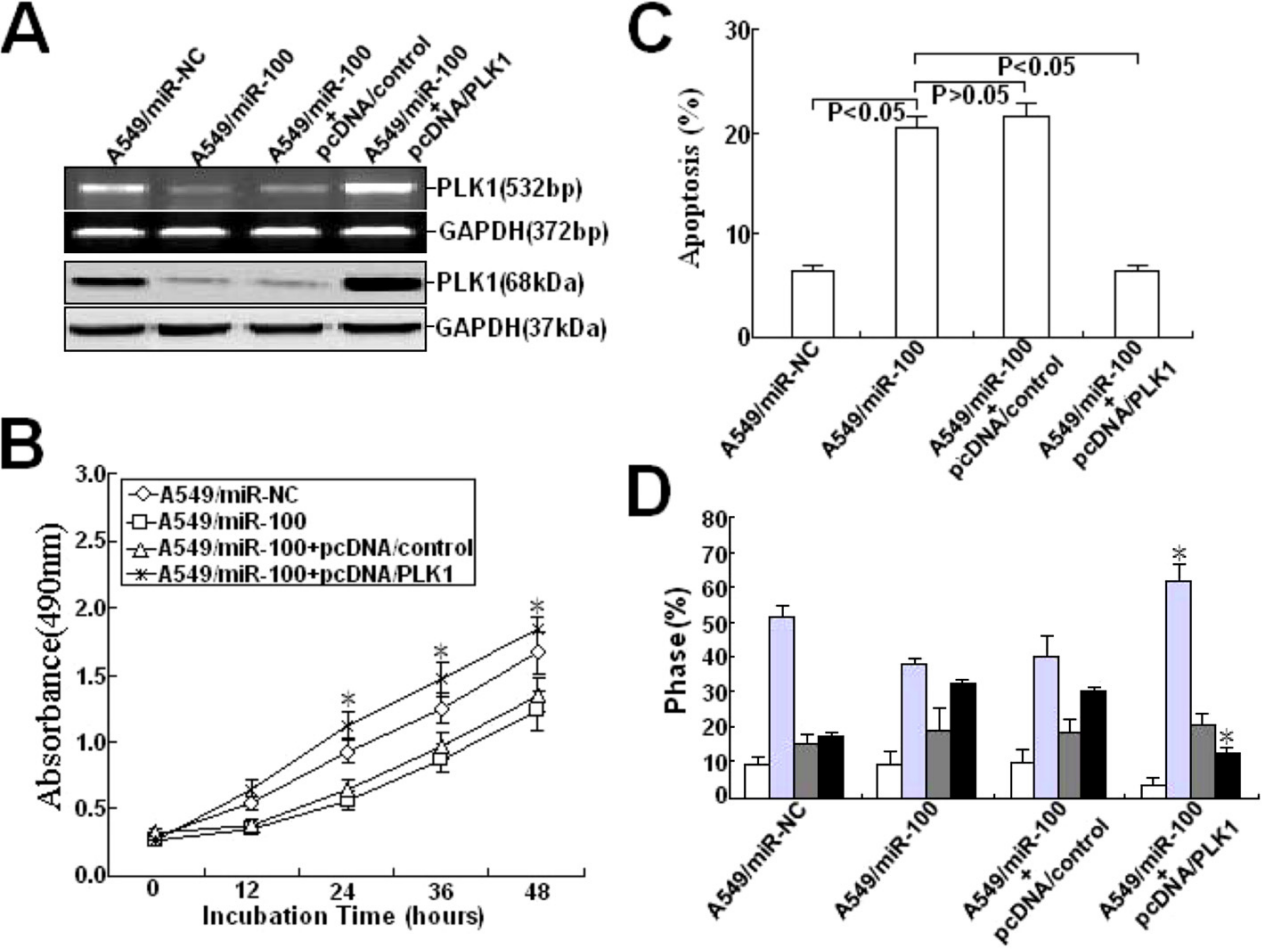
**DNA vector-mediated PLK1 overexpression could partially rescue the effects of miR-100 mimics on malignant phenotypes of A549 cells.** (**A**) RT-PCR and Western Blot analysis of PTEN mRNA and protein expression in A549/miR-NC, A549/miR-100 or A549/miR-100 co-transfected with pcDNA/control or pcDNA/PLK1. (**B**) MTT analysis of growth in A549/mi-NC, A549/miR-100 or A549/miR-100 co-transfected with pcDNA/control or pcDNA/PLK1. (**C**) Hoechst staining analysis of apoptosis in A549/miR-NC, A549/miR-100 or A549/miR-100 co-transfected with pcDNA/control or pcDNA/PLK1. The percentage of Hoechst-positive nuclei per optical field (at least 50 fields) was counted. (**D**) Flow cytometric analysis of cell cycle distribution in A549/miR-NC, A549/miR-100 or A549/miR-100 co-transfected with pcDNA/control or pcDNA/PLK1. * indicates *P* < 0.05. The data are expressed as the mean value ± SEM of the results obtained from three independent experiments. Corresponding *P* values analyzed by ANNOVA tests are indicated.

48h after pcDNA/PLK1 or pcDNA/control vector was transfected into A549/miR-100 cells, the expression levels of PLK1 mRNA and protein were determined. As shown in Figure 6A, pcDNA/PLK1 could rescue the decreased mRNA and protein expression in A549/miR-100 cells. Also, pcDNA/PLK1 could partially reverse growth inhibition and apoptosis enhancement of A549/miR-100 cells (Figure 6B-C). Moreover, we found that pcDNA/PLK1-mediated overexpression of PLK1 could partially reverse the G_2_/M phase cell cycle arrest of A549/miR-100 cells (Figure 6D). These results suggested that overexpression of PLK1 could rescue the effects of ectopic miR-100 on phenotypes of NSCLC cells.

### MiR-100 expression was inversely correlated with PLK1 mRNA expression in NSCLC tissues

**Figure 7:**
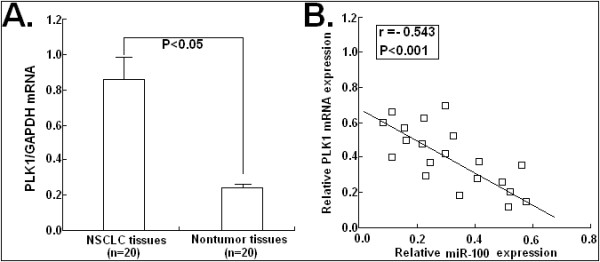
**PLK1 was significantly upregulated in NSCLC tissues and inversely correlated with miR-100 expression.** (**A**) The averaged mRNA level of PLK1 in NSCLC tissues (0.85 ± 0.15) was significantly higher than that in corresponding nontumor tissues (0.23 ± 0.06). GADPH was used as an internal control. (**B**) A statistically significant inverse correlation between miR-100 and PLK1 mRNA levels in 20 cases of NSCLC tissues (Spearman’s correlation analysis, r = −0.543; *P* < 0.001). Corresponding *P* values analyzed by a *t*-test or Spearman correlation test are indicated.

Then, semi-quantitative RT-PCR assay was performed to detect PLK1 mRNA expression in 10 NSCLC tissues and corresponding nontumor tissues. As shown in Figure 7A, the relative mRNA expression level of PLK1 in NSCLC tissues (0.85 ± 0.15) was significantly higher than that in corresponding nontumor tissues (0.23 ± 0.06; *P* < 0.05). When the levels of PLK1 mRNA were plotted against miR-100 expression, a significant inverse correlation was observed (r = −0.543; *P* < 0.001; Figure 7B). Thus, these data further support that downregulation of miR-100 was inversely correlated with upregulation of PLK1 in NSCLC tissues.

## Discussion

Previously, we have reported that PLK1 is overexpressed in human NSCLC tissues and the overexpression of PLK1 was correlated with poor prognosis and malignant phenotypes of NSCLC patients. However, the molecular mechanisms of PLK1 overexpression in NSCLC are still unclear. MiRNAs constitute a large family of small, approximately 21-nucleotide-long, non-coding RNAs that have emerged as key post-transcriptional regulators of gene expression, and miRNAs have been predicted to control the activity of approximately 30% of all protein-coding genes [18]. By base pairing to mRNAs, microRNAs mediate translational repression or mRNA degradation [19]. By performing in-silico screening using TargetScan, we found that the 3’-UTR of PLK1 gene contained binding sites for miR-100 with reasonable scores. In the present study, we showed that downregulation of miR-100 might play critical roles in the formation of malignant phenotypes by posttranscriptionally regulating PLK1 expression.

Up to date, increasing evidence shows that the dysregulation of miRNAs is correlated with tumor initiation and progression, suggesting that miRNAs may act as tumour suppressor genes or oncogenes [20,21]. Recent studies have shown that not only can miRNAs be used to sub-classify NSCLCs but specific miRNA profiles may also predict prognosis and disease recurrence in early-stage NSCLCs [22,23]. Since Takamizawa’ et al. firstly reported that reduced expression of the let-7 microRNAs in human lung cancers was found to be correlated with shortened postoperative survival of patients [24], other miRNAs were also found to be correlated with prognosis of NSCLC patients [25,26]. In our previous study, we also found that serum miR-21 expression might be useful as a prognostic marker for NSCLC patients [27]. With the development of research for experiment, dysregulation of miRNAs were reported to affect growth, apoptosis and chemo- or radioresistance of NSCLC cells [28]. Xiong and his colleagues found that microRNA-7 inhibited the growth of human non-small cell lung cancer A549 cells through targeting BCL-2 [29]. Moreover, miRNA-145 was found to inhibit non-small cell lung cancer cell proliferation by targeting c-Myc [30]. In our previous studies, we have shown that miR-451 functions as tumor suppressor in NSCLC by targeting RAB14 gene [31]. Meanwhile, we also found that the level of miR-451 could affect the sensitivity of NSCLC cells to cisplatin [32]. In other researches, ectopic expression of miR-200c alters expression of EMT proteins, sensitivity to erlotinib, and migration in lung cells [33]. The association of dysregulated miRNAs with angiogenesis and metastasis of NSCLC cells was reported. Donnem and his colleagues showed that several angiogenesis-related miRNAs (miR-21, miR-106a, miR-126, miR-155, miR-182, miR-210 and miR-424. miR-155) which were correlated significantly with fibroblast growth factor 2 (FGF2), are significantly altered in NSCLC [34]. MicroRNA-328 along with other miRNAs was found to be associated with (non-small) cell lung cancer (NSCLC) metastasis and mediates NSCLC migration [35]. Although miR-100 has been found to function as a tumor suppressor in nasopharyngeal cancer, epithelial ovarian cancer, bladder cancer and acute myeloid leukemia [36,37], the expression of miR-100 and its roles in NSCLC development are unknown.

In the present study, we firstly found that miR-100 was significantly lower in NSCLC tissues than in corresponding nontumor tissues. Then, we analyzed the association of downregulated miR-100 with clinicopathologic factors of NSCLC patients. By statistical analysis, we found that miR-100 expression was significantly correlated with clinical stage, tumor classification and lymph node metastasis of NSCLC patients, suggesting that low miR-100 expression might play roles in NSLC progression. The disease-free survival showed between NSCLC patients with low miR-100 and those with high miR-100, but the overall survival of patients with high miR-100 was higher than that of patients with low miR-100. Furthermore, multivariate analysis using the Cox proportional hazard model indicated that miR-100 expression was an independent prognostic factor for NSCLC patients. Functional experiments showed that upregulation of miR-100 could inhibit growth of NSCLC cells, which might be apoptosis enhancement and cell cycle arrest in G_2_/M stage. Sequence analyses revealed that the 3’-UTR of PLK1 mRNA contains a putative site partially complementary to miR-100. By firefly luciferase activity assay, miR-100 could inhibit luciferase activity in the PLK1 WT but had no effect in the mutant construct. Meanwhile, miR-100 mimics or inhibitor could lead to the decreased or increased PLK1 expression in NSCLC at both transcriptional and translational levels. By functional analysis, it was shown that siRNA-mediated PLK1 downregulation could mimic the effects of miR-100 mimics on phenotypes of NSCLC cells and overexpression of PLK1 could partially reverse miR-100 mimics-induced phenotypical changes in NSCLC cells. Additionally, miR-100 expression was inversely correlated with PLK1 mRNA expression in NSCLC tissues. From these data, PLK1 is a direct and functional target gene in NSCLC. While a single miRNA can target many genes, multiple miRNAs can regulate a single gene. In acute myeloid leukemia, RBSP3 (a phosphatase-like tumor suppressor) has been validated as a bona fide target of miR-100 [37]. Zheng and his colleagues revealed a new pathway that miR-100 regulates G_1_/S transition and S-phase entry and blocks the terminal differentiation by targeting RBSP3, which promoted cell proliferation. However, the functions of RBSP3 and its correlation with posttranscriptional regulation of miR-100 in NSCLC are unclear and remains to be elucidated in future research.

## Conclusions

Our results showed that low miR-100 might be a poor prognostic factor for NSCLC patients. As the number of patients in the present study is small, further study of a larger case population is necessary to confirm the clinical significance of miR-100 expression in NSCLC. Also, overexpression of miR-100 could inhibit growth, enhance apoptosis and induce cell cycle arrest in G_2_/M stage, which is possibly owing to increased apoptosis associated with downregulation of PLK1 expression. This raises the possibility that anti-miR-100 may have potential therapeutic value for NSCLC. While the goal of this study was to better understand miR-100 function in NSCLC, future research is required to address the therapeutic potential of modulating miR-100.

## Abbreviations

miRNA: microRNA; Ct: Cycle threshold; DMEM: Dulbecco’s modified Eagle’s medium; NSCLC: Non-small cell lung cancer; MTT: 3-(4,5-dimethylthazol-2-yl)-2,5-diphenyltetrazolium bromide; PLK1: Polo-like kinase 1; RT: Reverse transcription; qRT-PCR: Quantitative real-time reverse transcription polymerase chain reaction; SEM: Standard error of the mean; RR: Relative ratio; 95% CI: 95% confidence interval; SCC: Squamous cell carcinoma; AD: Adenocarcinoma.

## Competing interests

The authors declare that they have no competing interests.

## Authors’ contributions

LJ, LZL, and SM were involved in the conception and design of the study. LJ, LZL, and SM were involved in the provision of study material and patients. SM and DW performed the data analysis and interpretation. LJ wrote the manuscript. WZX approved the final version. All authors read and approved the final manuscript.

## Pre-publication history

The pre-publication history for this paper can be accessed here:

http://www.biomedcentral.com/1471-2407/12/519/prepub

## Supplementary Material

Additional file 1 Table S1. Association between miR-100 expression and clinicopathological features of NSCLC patients. (DOC 45 kb) Click here for file
