# Supplementary material for: MicroRNA-100 is a potential molecular marker of non-small cell lung cancer and functions as a tumor suppressor by targeting polo-like kinase 1
Source: BMC Cancer. 2012 Nov 14;12:519. doi: 10.1186/1471-2407-12-519 (PMC3521172; doi:10.1186/1471-2407-12-519)
Supplement: Additional file 1 — Table S1. Association between miR-100 expression and clinicopathological features of NSCLC patients. (DOC 45 kb) [file 1471-2407-12-519-S1.doc]

**Supplementary Table 1**

**Association between miR-100 expression and clinicopathological**

**features of NSCLC patients**

| Factors | MiR-100 expression | | *P*-value |
| --- | --- | --- | --- |
|  | High (n=46) | Low (n=64) |  |
| Sex |  |  | 0.488 |
| Male | 31 | 39 |  |
| Female | 15 | 25 |  |
| Age (years) |  |  | 0.583 |
| ≤60 | 22 | 34 |  |
| >60 | 24 | 30 |  |
| Smoking |  |  | 0.359 |
| Nonsmoker | 22 | 25 |  |
| Smoker | 24 | 39 |  |
| Histological type |  |  | 0.871 |
| SCC | 33 | 45 |  |
| AD | 13 | 19 |  |
| Clinical stage |  |  | 0.005 |
| I/II | 29 | 23 |  |
| III | 17 | 41 |  |
| Tumor classification |  |  | 0.013 |
| T_1_/T_2_ | 26 | 21 |  |
| T_3_/T_4_ | 20 | 43 |  |
| Lymph node metastasis |  |  | 0.001 |
| N_0_ | 30 | 31 |  |
| N_1+2_ | 16 | 33 |  |

SCC: squamous cell cancer; AD: adenocarcinoma
